# CRACM/Orai ion channel expression and function in human lung mast cells

**DOI:** 10.1016/j.jaci.2012.01.070

**Published:** 2012-06

**Authors:** Ian Ashmole, S. Mark Duffy, Mark L. Leyland, Valerie S. Morrison, Malcolm Begg, Peter Bradding

**Affiliations:** a Department of Infection, Immunity and Inflammation, Institute for Lung Health, University of Leicester, Leicester, United Kingdom; b Department of Biochemistry, University of Leicester, Leicester, United Kingdom; c Respiratory Therapy Area Unit, GlaxoSmithKline, Stevenage, United Kingdom

**Keywords:** CRACM, Orai, Ca^2+^, asthma, mast cell, histamine, leukotriene C_4_, cytokine, GSK-7975A, Synta-66, [Ca^2+^]_i_, Intracellular free Ca^2+^, CRAC, Ca^2+^ release–activated Ca^2+^, DMSO, Dimethyl sulfoxide, HLMCs, Human lung mast cells, LTC_4_, Leukotriene C_4_, MC, Mast cell

## Abstract

**Background:**

Influx of extracellular Ca^2+^ into human lung mast cells (HLMCs) is essential for the FcεRI-dependent release of preformed granule-derived mediators and newly synthesized autacoids and cytokines. However, the identity of the ion channels underlying this Ca^2+^ influx is unknown. The recently discovered members of the CRACM/Orai ion channel family that carries the Ca^2+^ release–activated Ca^2+^ current are candidates.

**Objectives:**

To investigate the expression and function of CRACM channels in HLMCs.

**Methods:**

CRACM mRNA, protein, and functional expression were examined in purified HLMCs and isolated human bronchus.

**Results:**

CRACM1, -2, and -3 mRNA transcripts and CRACM1 and -2 proteins were detectable in HLMCs. A CRACM-like current was detected following FcεRI-dependent HLMC activation and also in HLMCs dialyzed with 30 μM inositol triphosphate. The Ca^2+^-selective current obtained under both conditions was blocked by 10 μM La^3+^ and Gd^3+^, known blockers of CRACM channels, and 2 distinct and specific CRACM-channel blockers—GSK-7975A and Synta-66. Both blockers reduced FcεRI-dependent Ca^2+^ influx, and 3 μM GSK-7975A and Synta-66 reduced the release of histamine, leukotriene C_4_, and cytokines (IL-5/-8/-13 and TNFα) by up to 50%. Synta-66 also inhibited allergen-dependent bronchial smooth muscle contraction in *ex vivo* tissue.

**Conclusions:**

The presence of CRACM channels, a CRACM-like current, and functional inhibition of HLMC Ca^2+^ influx, mediator release, and allergen-induced bronchial smooth muscle contraction by CRACM-channel blockers supports a role for CRACM channels in FcεRI-dependent HLMC secretion. CRACM channels are therefore a potential therapeutic target in the treatment of asthma and related allergic diseases.

Mast cells play a major role in the pathophysiology of asthma and related allergic diseases such as rhinitis, urticaria, and anaphylaxis.^1^ Allergens and many nonimmunological stimuli activate complex signaling cascades in mast cells that lead to the secretion of a plethora of autacoid mediators, cytokines, and proteases.^1^ Excess release of these mediators contributes to complex immunopathologies and symptoms. Current putative clinical inhibitors of human mast cell (MC) mediator release, such as cromoglycate and β_2_-adrenoceptor agonists, are ineffective in some tissues such as the skin,^2^ and in lung they show weak activity and/or rapid tachyphylaxis/desensitization to the effects both *in vitro* and *in vivo*.^2-5^ A novel potent inhibitor of mast cell secretion that maintains its activity on chronic administration would therefore be of great benefit for the treatment of asthma and allergy.

Ion channels are emerging as attractive targets for the functional modulation of inflammatory and structural nonexcitable cells.^6,7^ Channels carrying Ca^2+^, K^+^, and Cl^−^ regulate diverse cell processes including secretion,^8^ proliferation,^9^ adhesion,^10^ and migration.^11^ Influx of extracellular Ca^2+^ is an essential requirement for the IgE-dependent release of both preformed (granule-derived) mediators and newly generated autacoids and cytokines from mast cells.^12^ Receptor-mediated signaling in many nonexcitable cells including mast cells initiates an initial rise in intracellular Ca^2+^ because of its release from endoplasmic reticulum stores. The resulting store depletion induces Ca^2+^ entry through the plasma membrane, a process termed store-operated Ca^2+^ entry.^13^ The Ca^2+^ current passing through the plasma membrane is known as the Ca^2+^ release–activated Ca^2+^ (CRAC) current, and it is believed to play a central role in many physiological processes such as gene transcription, proliferation, and cytokine release.^13,14^ The Ca^2+^ release–activated Ca^2+^ current has been well characterized electrophysiologically in several cells including rodent mast cells,^15^ and the molecular components of the CRAC channel have been recently identified. STIM1 senses the endoplasmic reticulum Ca^2+^ concentration and transmits this information to the CRAC-channel pore.^16^ CRACM1 (also known as Orai1) was subsequently identified as the Ca^2+^-selective pore-forming protein in the plasma membrane.^17-20^ Mammalian cells express 2 further homologs—CRACM2 and CRACM3^21^—which also carry CRAC currents but exhibit distinct functional properties.^21^ CRACM1 may form heterodimeric channels with CRACM2 and CRACM3.^21^

Studies in a CRACM1 knockout mouse suggested that CRACM1 function is essential for mast cell degranulation, leukotriene C_4_ (LTC_4_) release, and TNFα production following IgE-dependent activation, while CRACM2 regulates T-cell responses.^22^ However, whether CRACM channels operate in human MCs is not known. Because of the profound differences between rodent mast cells and human MCs in terms of pharmacology, mediator content, immunological responsiveness, and ion channel expression,^23^ it cannot be assumed that findings regarding Ca^2+^ channels in rodent mast cells can be extrapolated to humans. In this study, we have therefore examined the expression and function of CRACM channels in human lung mast cells (HLMCs).

## Methods

Full experimental details are provided in the Methods section in this article’s Online Repository at www.jacionline.org.

### Human MC purification and cell culture

All human subjects gave written informed consent, and the study was approved by the Leicestershire Research Ethics Committee. HLMCs were purified from macroscopically normal human lung (n = 11 donors) obtained within 1 hour of resection for lung cancer as described previously.^24^ Final HLMC purity was more than 99%, and viability was more than 97%. HLMCs were cultured as described previously.^25^

The human MC line HMC-1 (a gift from Dr J. Butterfield, Mayo Clinic, Rochester, Minn) was cultured in Iscove’s modified Dulbeccos’s medium as described previously.^24^ HEK293 cells were cultured in Dulbecco modified Eagle medium (Invitrogen, Paisley, United Kingdom) containing 10% FCS.

### RT-PCR and quantitative RT-PCR

RT-PCR and quantitative RT-PCR were used to examine CRACM mRNA expression in HLMCs. Full details including primer sequences are provided in this article’s Online Repository at www.jacionline.org.

### Analysis of CRACM protein expression

Analysis of CRACM protein expression was undertaken by using Western blot. Full experimental details and information on the antibodies used are provided in this article’s Online Repository at www.jacionline.org.

### Patch-clamp electrophysiology

The whole-cell variant of the patch-clamp technique was used as described previously.^26^ Currents in some experiments were also evoked by using a ramp protocol consisting of a continuous voltage ramp from −120 to +120 mV. Further details are provided in this article’s Online Repository at www.jacionline.org.

The CRACM-channel blockers GSK-7975A and Synta-66^27^ (gifts from GlaxoSmithKline, Stevenage, United Kingdom), Gd^3+^, and La^3+^ were added directly to the recording chamber as required. GSK-7975A is compound 36 from patent WO 2010/1222089.

### Ca^2+^ imaging

Changes in intracellular-free Ca^2+^ ([Ca^2+^]_i_) were monitored fluorometrically by use of the Ca^2+^-sensitive probe Fura-2, as described previously.^8^ Baseline measurements of HLMC [Ca^2+^]_i_ were recorded as the mean of the 6 values preceding the addition of an anti-FcεRIα antibody (Fisher Scientific, Loughborough, United Kingdom). The postactivation value of [Ca^2+^]_i_ was recorded as the mean of the 6 recordings taken immediately after the point of inflection following the rapid rise in [Ca^2+^]_i_.

### HLMC activation for mediator release

Experiments were performed at 37°C. For the analysis of histamine and LTC_4_ release, 2 × 10^4^ HLMCs in 80 μL were added to a 96-well V-bottom plate in triplicate, immediately followed by 10 μL of 10 times the final concentration of CRACM-channel blocker or dimethyl sulfoxide (DMSO) control. Plates were incubated for 10 minutes before the activation of cells by the addition of 10 μL of 10 times anti-FcεRIα antibody (final dilution 1:300). Plates were incubated for 30 minutes and centrifuged, and the supernatant was stored at −20°C for the measurement of mediator content. Control cell pellets were lyzed in ultrapure water for the determination of total histamine content. For the analysis of cytokine release, the final cell concentration was 0.666 × 10^6^ cells/mL, and IgE-sensitized cells were activated with anti-IgE (Hybridoma Reagents Laboratory, Baltimore, Md; final concentration 2 μg/mL) for 16 hours before harvesting the culture supernatant.

### Mediator assays

Histamine was measured by radioenzymatic assay and LTC_4_ by ELISA as described previously.^24,26^ The cytokines IL-5, IL-8, IL-13, and TNFα were measured blind by using the Meso Scale Discovery platform.

### Allergen-induced bronchial smooth muscle contraction in isolated human bronchus

Lung tissue was obtained postmortem. Airways were dissected free of lung parenchyma and adjoining blood vessels. Secondary and tertiary bronchi, with cartilaginous walls and diameters of 3 to 10 mm, were cut spirally into strips 3 to 5 mm wide and then cut into pieces 10 to 15 mm long. The strips were passively sensitized overnight at room temperature (21°C) in atopic serum (20% v/v) in Krebs buffer. Before use, sensitized tissues were washed free of serum. Tissues were mounted under 1.5 g of resting tension in an immersion organ bath, maintained in oxygenated Krebs buffer solution at 37°C, and allowed to equilibrate for 30 to 45 minutes with 2 washes and retensioning if required.

Two preliminary “priming” contractions to 10 μM methacholine (Sigma, Poole, United Kingdom) were performed. The tissue was then incubated with Synta-66 (10 μM) or DMSO control (0.1% final concentration) for 1 hour. Grass allergen (Six grass mix, ALK-Abelló, Hungerford, United Kingdom) was then added cumulatively (0.1-30 U/mL final concentration), with contractions measured in milligrams tension. This was followed by a final measurement of contraction to 10 μM methacholine. Data were expressed as percentage of the initial 10 μM methacholine contraction.

## Results

### HMCs express CRACM1, -2, and -3 mRNAs

**Fig 1:**
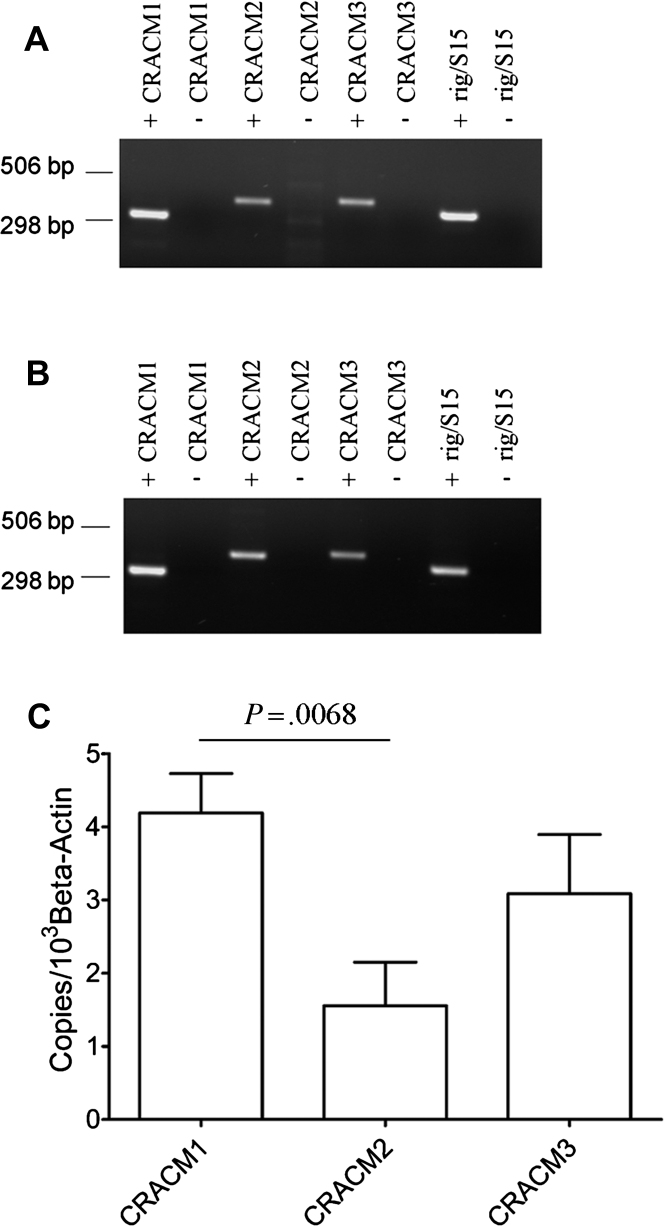
HLMCs express CRACM1, CRACM2, and CRACM3 mRNAs. RT-PCR of CRACM1, -2, and -3 and rig/S15 mRNA transcripts using RNA purified from HLMCs **(A)** and from HMC-1 cells **(B)** in the presence (+) or absence (−) of reverse transcriptase. **C,** Quantitative RT-PCR of CRACM mRNA transcripts in HLMCs relative to β-actin transcripts (mean ± SEM; n = 6-8). CRACM1 versus CRACM2, *P* = .0068.

RT-PCR and quantitative RT-PCR experiments were performed to determine the expression of CRACM-channel mRNAs in HLMCs. Robust expression of CRACM1 (n = 8 donors), CRACM2 (n = 6 donors), and CRACM3 (n = 6 donors) was observed in all donors examined and also in HMC-1 cells (Fig 1, *A* and *B*). Normalizing the amount of each CRACM transcript to the amount of either β-actin mRNA (Fig 1, *C*) or 18S RNA (data not shown) revealed CRACM1 to be the most abundant of the 3 CRACM transcripts expressed in HLMCs. CRACM2 mRNA was the least abundantly expressed, with levels of CRACM3 being intermediate. Only the difference in levels of expression between CRACM1 and CRACM2 reached statistical significance (*P* = .0068; overall comparing all 3 transcripts: *P* = .0299). Similar relative amounts of CRACM transcripts were observed in HMC-1 cells (data not shown).

### HLMCs express CRACM1 and -2 proteins

**Fig 2:**
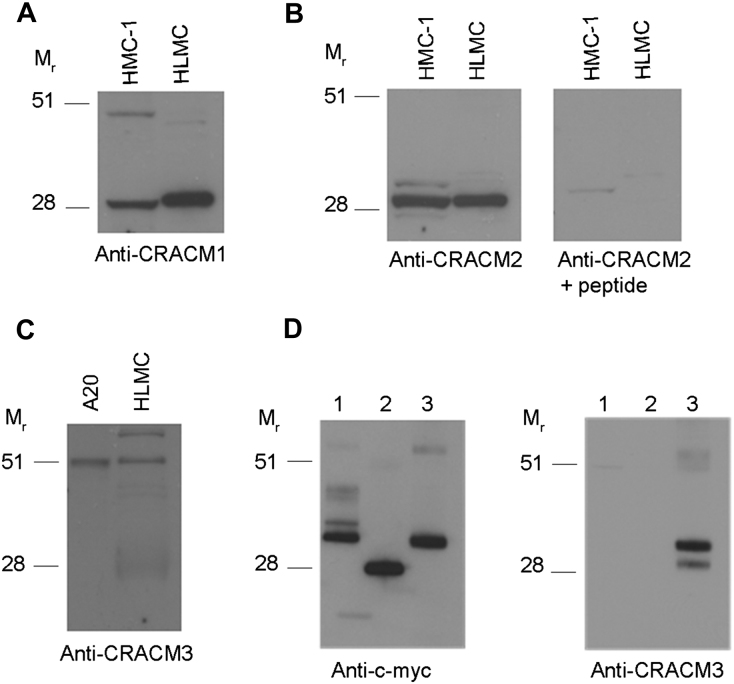
HLMCs express CRACM1 and CRACM2 proteins. **A, B,** and **C,** Representative Western blots using the indicated antibodies and lysates of HMC-1 and HLMCs (Fig 2, *A* and *B)* or A-20 cells and HLMCs (Fig 2, *C*). Three independent mast cell lysates were analyzed. **D,** Western blots using the indicated antibodies and lysates of HEK293 cells transiently transfected with vectors directing the expression of CRACM1-Myc (lane 1), CRACM2-Myc (lane 2), or CRACM3-Myc (lane 3).

Western blotting of 3 HLMC lysates from 3 independent donors revealed the presence of bands close to the predicted molecular weight of CRACM1 (32.7 kDa) and CRACM2 (28.6 kDa) (Fig 2, *A*). A blocking peptide for CRACM2 was available and inhibited CRACM2 staining (Fig 2, *B*). Blotting for CRACM3 in HLMCs failed to convincingly demonstrate the presence of band(s) close to the predicted molecular weight of CRACM3 (31.5 kDa). A higher molecular weight band was however identified (Fig 2, *C*). The Western blotting of whole-cell lysates of HEK293 cells transiently transfected with a construct directing the expression of the myc epitope–tagged CRACM3 protein did reveal a band of the expected size, indicating that the band identified in HLMCs is likely to be nonspecific (Fig 2, *D*).

### HLMCs express CRACM currents following store depletion with IP_3_

**Fig 3:**
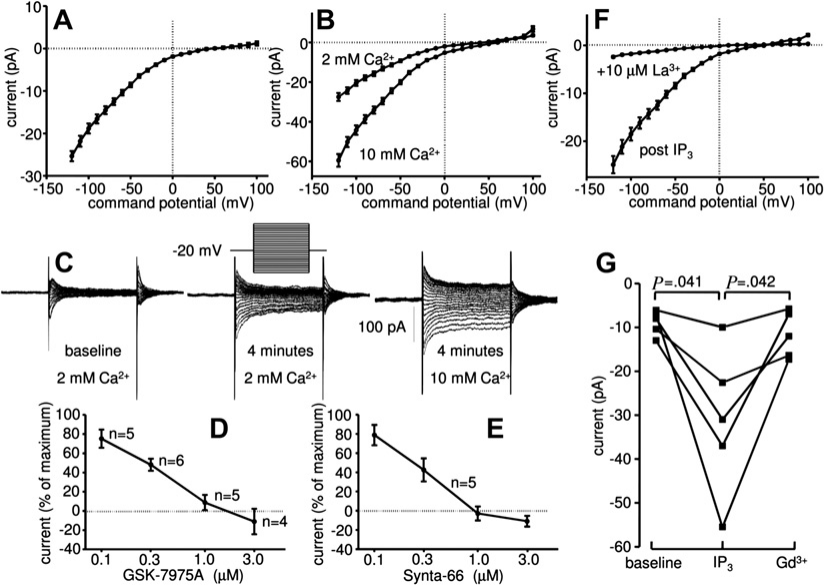
HLMCs express CRACM-channel currents when dialyzed with IP_3_. Subtracted whole-cell patch-clamp current-voltage (I-V) curves from HLMCs 4 minutes after dialysis with IP_3_ in 2 mM external Ca^2+^ (mean ± SEM; n = 42 cells) **(A)** and in 2 mM and then 10 mM external Ca^2+^ (mean ± SEM; n = 6) **(B)**. **C,** Representative raw current traces from a single cell in the presence of IP_3_ (voltage protocol shown inset). Inhibition of IP_3_-dependent whole-cell current by **(D)** GSK-7975A and **(E)** Synta-66 (n = 4-6) (a negative value implies a contribution from CRACM channels to the baseline whole-cell current) and **(F)** 10 μM La^3+^ (n = 12) and **(G)** 10 μM Gd^3+^.

To investigate whether HLMCs expressed a Ca^2+^ current induced by store depletion, cells were dialyzed with 30 μM IP_3_. This resulted in the development of an inwardly rectifying current with the electrophysiological features of CRAC currents, the current carried by CRACM channels (Fig 3, *A*) in 90% of the cells tested. The subtracted IP_3_-dependent current (IP_3_ whole-cell current minus baseline whole-cell current) peaked at a mean of 25.4 ± 1.2 pA at −120 mV with a reversal potential of 49.9 ± 1.3 mV within 4 minutes of achieving the whole-cell configuration (n = 42 cells from 9 donors, *P* < .0001, compared with baseline for both current and reversal potential) (Fig 3, *A*). The IP_3_-dependent current was increased by 32.0 ± 3.2 pA by increasing extracellular Ca^2+^ to 10 mM (n = 6 cells; *P* = .004) (Fig 3, *B*), with reversal potential shifting to 59.6 ± 4.4 mV (*P* = .009). Inspection of the raw current obtained during voltage steps showed typical features of CRACM channels with current evoked immediately following each voltage step, and with mild decay over 100 ms (Fig 3, *C*). Similar results were seen by using voltage ramps (data not shown). Further pharmacological analysis of the current showed that it was blocked dose dependently by GSK-7975A (IC_50_ of 3.4 × 10^−7^ mol/L) (Fig 3, *D*) and Synta-66 (IC_50_ of 2.5 × 10^−7^ mol/L) (Fig 3, *E*). This is consistent with the blocking of CRACM channels by these compounds in previous studies^27^ (and unpublished data). In addition, the IP_3_-induced current was blocked by 10 μM La^3+^ (Fig 3, *F*) and 10 μM Gd^3+^ (Fig 3, *G*), consistent with the properties of CRACM channels.^17,22,28^ CRACM currents did not develop in control cells in the absence of IP_3_ (n = 9).

### HLMCs develop CRACM currents following activation with anti-IgE

**Fig 4:**
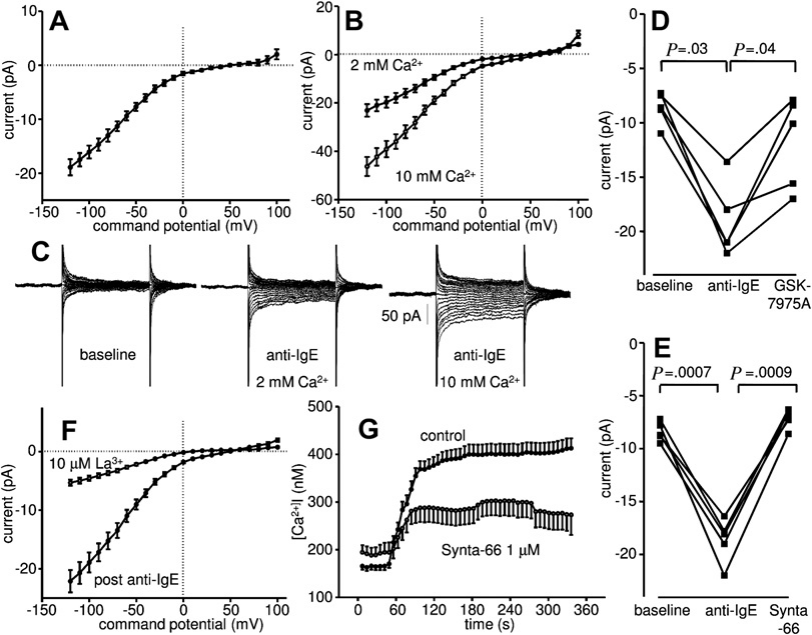
HLMCs express CRACM-channel currents following FcεRI-dependent activation. Whole-cell patch-clamp current-voltage (I-V) curves of HLMCs within 4 minutes of FcεRI-dependent activation in **(A)** 2 mM external Ca^2+^ (mean ± SEM; n = 37 cells) and **(B)** 2 mM and then 10 mM external Ca^2+^ (mean ± SEM; n = 7). **C,** Representative raw current following FcεRI-dependent activation. Inhibition of IgE-dependent CRACM currents by **(D)** 1 μM GSK-7975A, **(E)** 1 μM Synta-66, and **(F)** 10 μM La^3+^ (n = 11 cells). **G,** Attenuation of the FcεRI-dependent increase in [Ca^2+^]_i_ in HLMCs by 1 μM Synta-66.

Cross-linking the high-affinity IgE receptor FcεRI with the addition of anti-IgE to the recording chamber induced the development of a current similar to that seen with IP_3_ in 90% of the HLMCs tested (Fig 4, *A*). The subtracted IgE-dependent current peaked at a mean of 18.9 ± 1.5 pA at −120 mV with a reversal potential of 49.1 ± 1.4 mV within 4 minutes of cell activation (n = 37 cells from 7 donors, *P* < .0001, compared with baseline for both current and reversal potential). The current was increased by 23.2 ± 3.9 pA by increasing extracellular Ca^2+^ to 10 mM (n = 7 cells; *P* = .003) (Fig 4, *B*), with a shift in reversal potential to 66.0 ± 3.9 mV (*P* = .005). Inspection of the raw current obtained during voltage steps again showed typical features of CRACM channels with current evoked immediately with each voltage step and with mild decay over 100 ms (Fig 4, *C*). Similar results were seen by using voltage ramps (data not shown). Further pharmacological analysis of the IgE-dependent current showed it was blocked by both 1 μM GSK-7975A (n = 5 cells; *P* = .04) (Fig 4, *D*) and 1 μM Synta-66 (n = 5 cells; *P* = .0009) (Fig 4, *E*). In addition, the IgE-dependent current was blocked by 10 μM La^3+^ (n = 11; *P* = .0003) (Fig 4, *F*). Taken together, these findings are consistent with the development of CRACM currents in HLMCs following IgE-dependent activation.

### CRACM-channel blockers attenuate IgE-dependent Ca^2+^ influx in HLMCs

Activation of HLMCs with anti-FcεRIα induced an acute increase in [Ca^2+^]_i_ followed by a plateau phase as described previously^8^ (Fig 4, *G*). In total, 111 of 118 (94%) cells responded. In the absence of CRACM-channel blockers, [Ca^2+^]_i_ increased from a mean baseline of 164.9 ± 9.0 to 375.6 ± 17.4 nM (n = 66 cells from 3 donors; *P* < .0001) following FcεRIα-dependent activation. In HLMCs from matched donors, [Ca^2+^]_i_ increased from a mean baseline of 191.7 ± 16.4 to 287.0 ± 25.7 nM (n = 52 cells from 3 donors; *P* < .0001) with 1 μM Synta-66. There was a significant difference in the absolute change in [Ca^2+^]_i_ in FcεRIα-activated control cells versus those activated in the presence of Synta-66 (*P* < .0001). In summary, 1 μM Synta-66 reduced the FcεRIα-dependent increase in [Ca^2+^]_i_ by 54.8% ± 9.1%.

### CRACM-channel blockers attenuate HLMC mediator release

**Fig 5:**
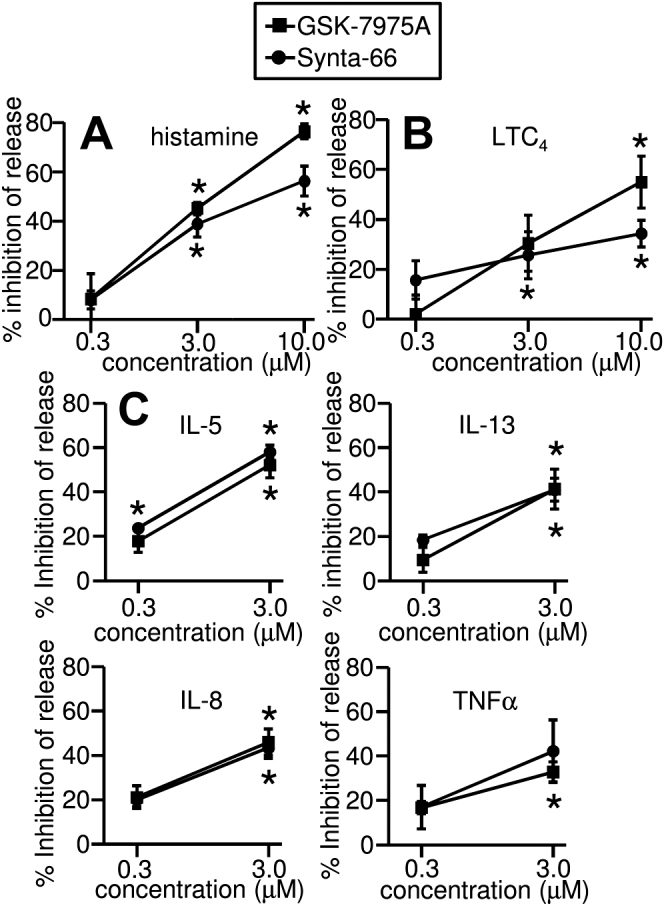
The CRACM-channel blockers GSK-7975A and Synta-66 inhibit HLMC histamine **(A)**, LTC_4_**(B)**, and cytokine release **(C)** dose-dependently following FcεRI-dependent activation. Mean ± SEM for percentage inhibition (n = 5 for histamine and LTC_4_, n = 3 for cytokines). *P* < .05 for all conditions except Synta-66 TNFα analyzed by repeated-measures ANOVA on raw data. ∗*P* < .05 compared with control using Bonferroni post hoc test.

Following activation with anti-FcεRIα in the presence of DMSO control, HLMCs released histamine (net 25.3% ± 4.8% of total histamine content, a marker of degranulation), 125.7 ± 32.5 ng/10^6^ cells of LTC_4_ (a marker of arachidonic acid metabolism), and IL-5 (505.1 ± 142.7 pg/10^6^ cells), IL-8 (8880 ± 3469 pg/10^6^ cells), IL-13 (140.5 ± 78.7 pg/10^6^ cells), and TNFα (618.3 ± 73.4 pg/10^6^ cells). Both GSK-7975A and Synta-66 dose dependently attenuated the release of these mediators (*P* < .05 by repeated-measures ANOVA for all drugs and mediators with the exception of TNFα inhibition by Synta-66; *P* = .087) (Fig 5, *A*-*C*). Net IgE-dependent histamine release was reduced by 45.2% ± 2.5% (n = 5; *P* < .0001) and 38.8% ± 5.4% (n = 5; *P* < .0001) in the presence of 3 μM GSK-7975A and 3 μM Synta-66, respectively. A similar degree of inhibition was seen with the release of LTC_4_ (Fig 5, *B*) and the above cytokines (Fig 5, *C*).

### CRACM blockade attenuates allergen-induced bronchial smooth muscle contraction in isolated human bronchus

**Fig 6:**
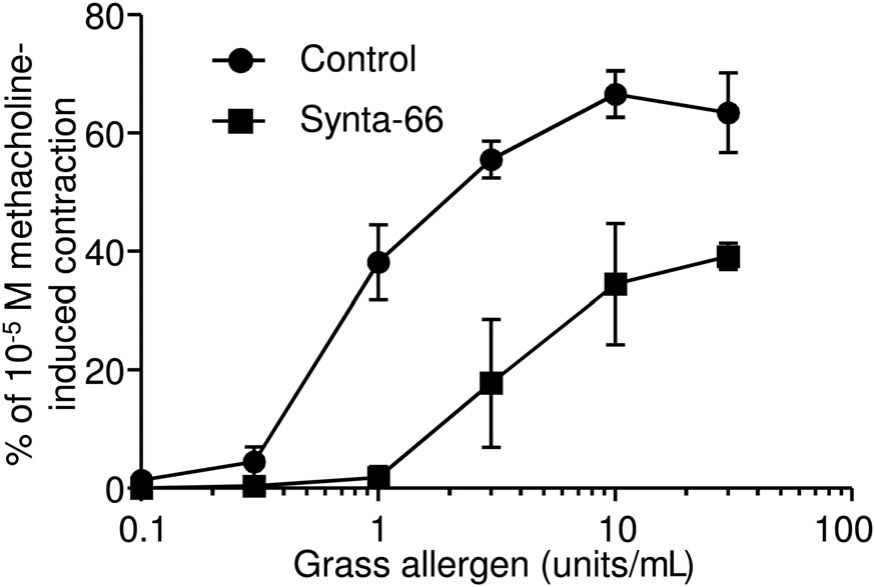
The CRACM-channel blocker Synta-66 attenuates allergen-dependent human bronchial smooth muscle contraction (n = 4). *P* = .02 for rightward shift in allergen EC_50_.

Allergen-induced bronchoconstriction was assessed in isolated human bronchus. The application of allergen induced a dose-dependent increase in bronchial smooth muscle contraction (Fig 6) (geometric mean EC_50_ in DMSO control 0.825 [95% CI 0.50-1.35] grass allergen units/mL; maximal response 63.6% ± 4.1% of that induced by 10 μM methacholine; n = 4). In the presence of Synta-66 10 μM, there was a rightward shift in the allergen dose-response curve (EC_50_ 4.14 [95% CI 1.72-9.96] grass allergen units/mL; *P* = .02). In 3 out of 4 experiments, there was a marked reduction in the maximal response (39.7% ± 7.0% of methacholine response for all data; *P* = .084). Synta-66 had no effect on methacholine-induced contraction (data not shown).

## Discussion

In spite of the absolute requirement for an influx of extracellular Ca^2+^ for the FcεRI-dependent release of preformed granule-derived mediators, newly generated leukotrienes and prostaglandins, and many cytokines in human MCs, the Ca^2+^ entry pathway has not been defined. Studies of knockout mice lacking CRACM1 function have shown that CRACM channels are essential for the influx of extracellular Ca^2+^ into rodent mast cells following their activation.^22^ In addition, indirect evidence has implicated CRACM channels as the means of Ca^2+^ influx in human MCs derived from nasal polyps.^29^ Here we show for the first time that HLMCs express CRACM1, -2, and -3 at the mRNA level, at least CRACM1 and -2 at the protein level, and following IgE-dependent activation, functional CRAC currents.

Our results are consistent with CRACM channels playing a role in the influx of extracellular Ca^2+^ into HLMCs following their activation. Two specific pharmacological blockers of CRACM channels—GSK-7975A and Synta-66^27^—reduced the increase in intracellular Ca^2+^ that occurs following ligation of FcεRIα and attenuated the release of histamine, LTC_4_, and several cytokines. The inhibition by these drugs occurred in the dose range of channel block demonstrated electrophysiologically. It takes approximately 5 to 10 times the IC_50_ of a channel blocker to inhibit 100% of the relevant channels. At 4 times the IC_50_, Synta-66 reduced FcεRI-dependent Ca^2+^ influx by 50% and at 10 times the IC_50_, both GSK-7975A and Synta-66 inhibited mediator release by up to 50%.

The biological relevance of these findings with respect to asthma is highlighted by the ability of Synta-66 to inhibit allergen-induced bronchial smooth muscle contraction in *ex vivo* passively sensitized bronchial tissue. The acute bronchoconstrictor smooth muscle response to allergen challenge is entirely dependent on the release of bronchospastic mediators from airway mast cells.^30^ In keeping with the attenuation of HLMC Ca^2+^ influx and mediator release observed with both Synta-66 and GSK-7975A, Synta-66 shifted the dose-response curve for allergen-dependent bronchial smooth muscle contraction 5-fold to the right and markedly reduced the maximal allergen-dependent response in 3 out of 4 donors. It should be noted that bronchial smooth muscle cells express CRACM1 and demonstrate store-operated Ca^2+^ currents,^31^ but it is unlikely that these currents in airway smooth muscle contribute to allergen-induced bronchoconstriction induced by mast cell mediators. This is because CRACM blockade had no effect on bronchial smooth muscle contraction induced directly by methacholine, which means that it is unlikely that it would inhibit the histamine and leukotriene-dependent contraction following allergen-dependent mast cell degranulation. Thus, the highly reproducible responses in both isolated HLMCs and tissue in the presence of CRACM-channel blockers suggests that the predominant site of activity of the CRACM inhibition in tissue is the mast cell.

Our results indicate that although important, CRACM channels may not be solely responsible for Ca^2+^ influx into activated HLMCs. The substantial residual histamine, LTC_4_, and cytokine secretion that we observe using high concentrations of blockers indicates that further Ca^2+^-permeable channels and/or receptors may play at least some role in Ca^2+^ influx into HLMCs. These results are in contrast to those from CRACM1 knockout mice where antigen-evoked Ca^2+^ influx into mast cells is reportedly reduced by 70% with the remaining Ca^2+^ influx being blocked by CRACM-channel inhibitors.^22^ Our results therefore highlight further the heterogeneity of mast cells from different species and underline the importance of studying human MCs rather than attempting to extrapolate results from rodent mast cells.

In addition to CRACM, mast cells express a number of other ion channels/receptors that may allow the entry of extracellular Ca^2+^. In rodents, the L-type voltage-gated Ca^2+^ channel Ca_v_1.2 may be involved in Ca^2+^ influx independent of endoplasmic reticulum Ca^2+^ store emptying following mast cell activation.^32^ However, we have never observed a Ca_v_-like current in HLMCs although these cells do express mRNA for Ca_v_3.3 and the α_2_δ_2_ subunit.^33^ Our laboratory has also shown that HLMCs express the P2X receptors P2X1, P2X4, and P2X7, which although acting as nonselective cation channels can produce significant Ca^2+^ influx in response to nucleotides such as ATP.^34^ Finally, much attention has been focused on the potential role of canonical transient receptor potential channels in Ca^2+^ entry following cell activation that function as nonselective cation channels able to pass Ca^2+^. The potential role of all these channels will require further investigation.

Our work provides strong evidence for the expression of both CRACM1 and CRACM2, with CRACM1 transcripts present in significantly higher amounts. To assess the contribution of each channel to HLMC Ca^2+^ entry will require the use of knockdown strategies and the use of dominant negative mutants in future work. In mouse mast cells CRACM1 dominates, while in mouse T cells CRACM2 expression is the highest and CRACM1 is dispensable for cell function.^22^ However, in human T cells, CRACM1 is essential for cell function, and its complete absence results in one form of hereditary severe combined immune deficiency.^17^ Interestingly, while the expression of wild-type CRACM1 in T cells from patients with severe combined immune deficiency fully restores the CRAC current, expression of either CRACM2 and/or CRACM3 is reported to have little or no effect,^35^ demonstrating that these channels have distinct roles.

Given the relative abundance of CRACM3 mRNA transcripts in HLMCs, we were surprised not to be able to demonstrate CRACM3 protein expression by Western blotting. It is possible that CRACM3 is more sensitive to proteolysis than are its homologs. Proteolysis has been noted as a problem in the analysis of the protein expression of other mast cell ion channels.^32^ However, we have also been unable to demonstrate the expression of CRACM3 by flow cytometry (data not shown), suggesting that if it is expressed as a protein, it is expressed in relatively very low amounts.

In conclusion, we have demonstrated the presence of functional CRACM channels in HLMCs. Treatment of HLMCs with CRACM-channel blockers reduced the release of mediators and cytokines; CRACM channels are therefore a potential therapeutic target in the treatment of asthma and related allergic diseases.

**Key messages**

• The Ca^2+^ influx pathway required for FcεRI-dependent HLMC mediator release is not known.
• HLMCs express CRACM ion channels that contribute to at least 50% of the Ca^2+^ influx required for FcεRI-dependent histamine, LTC_4_, and cytokine release, and allergen-induced bronchial smooth muscle contraction.
• CRACM channels are a potential therapeutic target in the treatment of asthma and related allergic diseases.

## Methods

### Human mast cell purification and cell culture

All human subjects gave written informed consent, and the study was approved by the Leicestershire Research Ethics Committee. HLMCs were purified from macroscopically normal human lung (n = 11 donors) obtained within 1 hour of resection for lung cancer as described previously.^E1^ Final HLMC purity was more than 99%, and viability was more than 97%. HLMCs were cultured as described previously.^E2^ Cells were sensitized with human myeloma IgE 2.5 μg/mL (Merck Bioscience Ltd, Nottingham, United Kingdom) as required.

The human MC line HMC-1 (a gift from Dr J. Butterfield, Mayo Clinic, Rochester, Minn) was cultured in Iscove’s modified Dulbeccos’s medium (Invitrogen, Paisley, United Kingdom) as described previously.^E1^ HEK293 cells were cultured in Dulbecco modified Eagle’s medium (Invitrogen) containing 10% FCS.

### RT-PCR and quantitative RT-PCR

Total RNA was isolated from cells by using an RNAqueous-4PCR kit (Applied Biosystems, Warrington, United Kingdom) according to the manufacturer’s instructions. HMC-1 cells (1 μg) or HLMCs (0.4-1 μg) of total RNA was used to generate cDNA by using random decamer primers and a Retroscript kit (Applied Biosystems). The following primer pairs were designed to amplify channel cDNA: CRACM1 5′GCCAGAGTTACTCCGAGGTG3 ′ and 5′TGACCGAGTTGAGATTGTGC3 ′, CRACM2 5′TCGACCCCTCTGCTCCTGCC3 ′ and 5′TGCGGGGACTCGCTGATGGA3 ′, and CRACM3 5′TCGGCCACGTACCGGGAGTT3′ and 5′GCCCAGGGCAGTGGAGAAGC3 ′. The predicted product sizes were 311, 390, and 405 bp respectively. Primers 5′TTCCGCAAGTTCACCTACC3 ′ and 5′CGGGCCGGCCATGCTTTACG3 ′ designed to amplify a 361-bp sequence from the small ribosomal subunit protein rig/S15 cDNA were used as a positive control. Pfu DNA Polymerase (Promega, Southampton, United Kingdom) was used for the PCR amplification.

For quantitative PCR, TaqMan probes for human CRACM1 (Hs00385627_m1), CRACM2 (Hs00259863_m1), and CRACM3 (Hs00752190_s1) were used together with TaqMan Gene Expression Master Mix (all Applied Biosystems). Reactions were run on a Stratagene Mx3000P (Agilent Technologies, Stockport, United Kingdom) real-time thermocycler. For some HLMC donors, limiting amounts of RNA precluded the analysis of CRACM1, -2, and -3 expression together in the same experiment. When this was the case, expression of 2 of the 3 CRACM transcripts was analyzed.

### Analysis of CRACM protein expression

Cells were lyzed in Radio-Immunoprecipitation Assay (RIPA) buffer (50 mM Tris HCl, pH 8.0, 150 mM NaCl, 1% NP-40, 0.5% sodium deoxycholate, 0.1% SDS) containing protease inhibitors. Proteins were separated on 12% Bis-Tris Nu-Page gels (Invitrogen) and then blotted onto polyvinyidene fluoride membranes. Membranes were blocked with 5% nonfat milk in PBS and then probed with rabbit polyclonal antibodies recognizing CRACM1 (Alomone Labs Ltd, Jerusalem, Israel), CRACM2 (Alomone Labs Ltd), CRACM3 (AbD Serotec, Kidlington, United Kingdom), or a mouse monoclonal anti-c-Myc antibody (clone 9E10, Sigma, Poole, United Kingdom). In addition, the anti-CRACM2 antibody was used following preincubation with its immunogenic (blocking) peptide. Blots were subsequently probed with goat antirabbit immunoglobulins-horseradish peroxidise secondary antibody (Dako, Cambridge, United Kingdom) or goat antimouse IgG-horseradish peroxidise (Santa Cruz Biotechnology, Inc, Heidelberg, Germany) as appropriate. Immunoreactive bands were visualized by using Pierce ECL Western Blotting Substrate (Fisher Scientific, Loughborough, United Kingdom).

For the expression of c-Myc epitope–tagged CRACM channels, full-length CRACM1 (accession no. NM_032790), CRACM2 (accession no. NM_032831), and CRACM3 (accession no. NM_152288) were amplified from cDNA generated from total RNA purified from a single HLMC donor using Pfu DNA polymerase. cDNAs were then cloned in frame immediately following the c-Myc epitope tag in vector pCruz Myc (Santa Cruz Biotechnology Inc). HEK293 cells were transiently transfected with vectors directing the expression of c-Myc epitope–tagged CRACM channels using GeneJuice transfection reagent (Merck Bioscience Ltd) and harvested for lysis 24 hours after transfection.

### Patch-clamp electrophysiology

The whole-cell variant of the patch-clamp technique was used.^E3,E4^ Patch pipettes were made from borosilicate fiber-containing glass (Clark Electromedical Instruments, Reading, United Kingdom), and their tips were heat polished, typically resulting in resistances of 4 to 6 MΩ. The standard pipette solution contained CsCl (140 mM), MgCl_2_ (2 mM), HEPES (10 mM), NaATP (2 mM), and GTP (0.1 mM), pH 7.3, with KOH. For IP_3_ experiments, 5 mM EGTA was also added to the pipette solution. For anti-IgE experiments, intracellular Ca^2+^ was buffered to 250 nM by using CaCl_2_ and ethyleneglycol-bis-(β-aminoethylether)-*N*,*N*,*N*′,*N*′-tetraacetic acid. The standard external solution contained NaCl (140 mM), KCl (5 mM), CaCl_2_ (2 mM), MgCl_2_ (1 mM), HEPES (10 mM), and glucose (5 mM), pH 7.3, with NaOH. For recording, mast cells were placed in 35-mm dishes containing standard external solution.

Whole-cell currents were recorded by using an Axoclamp 200A amplifier (Axon Instruments, Foster City, Calif), and currents usually evoked by applying voltage commands to a range of potentials in 10-mV steps from a holding potential of −20 mV. The currents were digitized (sampled at a frequency of 10 kHz), stored on computer, and subsequently analyzed by using pClamp software (Axon Instruments). Capacitance transients were minimized by using the capacitance neutralization circuits on the amplifier. Correction for series resistance was not routinely applied. Experiments were performed at 27°C, temperature being controlled by a Peltier device. Experiments were performed with a perfusion system (Automate Scientific, Inc, San Francisco, Calif) to allow solution changes, although drugs were added directly to the recording chamber. Currents in some experiments were also evoked by using a ramp protocol consisting of a continuous voltage ramp from −120 to +120 mV.

The CRACM-channel blockers GSK-7975A and Synta-66^E5^ (gifts from GlaxoSmithKline, Stevenage, United Kingdom), Gd^3+^ (gadolinium(III) chloride), and La^3+^ (lanthanum(III) chloride) were added directly to the recording chamber as required. GSK-7975A is compound 36 from patent WO 2010/1222089.

### Ca^2+^ imaging

Changes in [Ca^2+^]_i_ were monitored fluorometrically by use of the Ca^2+^-sensitive probe Fura-2. Cells were loaded with Fura-2 (Molecular Probes, Eugene, Ore) by incubation in normal physiological saline solution (130 mM NaCl, 5.6 mM KCl, 1 mM MgCl_2_, 2 mM CaCl_2_, 11 mM glucose, 10 mM HEPES, pH 7.4, with NaOH) containing 2 μM fura-2-acetoxymethyl ester (Fura-2-AM) for 30 minutes at room temperature and then washed for 30 minutes. A coverslip with attached cells was mounted in a perfusion chamber at 37°C. The single-cell recording system has been described previously.^E2^ Data acquisition occurred at a rate of one dual-wavelength image every 6 seconds as the 340/380 nm ratio. This was converted to [Ca^2+^]_i_ by using a commercially available calibration kit (Molecular Probes). Drugs were added to the dish as required. Baseline measurements of HLMC [Ca^2+^]_i_ were recorded as the mean of the 6 values preceding the addition of an anti-FcεRIα antibody (Fisher Scientific). The postactivation value of [Ca^2+^]_i_ was recorded as the mean of the 6 recordings taken immediately after the initial peak.

### HLMC activation for mediator release

Experiments were performed at 37°C. For the analysis of histamine and leukotriene release, 2 × 10^4^ HLMCs in a volume of 80 μL were added to a 96-well V-bottom plate in triplicate, immediately followed by 10 μL of 10 times the final concentration of CRACM-channel blocker or DMSO control. Plates were incubated for 10 minutes before the activation of cells by the addition of 10 μL of 10 times anti-FcεRIα antibody (final dilution of antibody 1:300). Plates were incubated for 30 minutes and centrifuged, and the supernatant was decanted and stored at −20°C for the measurement of mediator content. Control cell pellets were lyzed in ultrapure water for the determination of total histamine content.

For the analysis of cytokine release, the final cell concentration was 0.666 × 10^6^ cells/mL, and IgE-sensitized cells were activated with anti-IgE (Hybridoma Reagents Laboratory, Baltimore, Md) (final concentration 2 μg/mL) for 16 hours before harvesting of the culture supernatant.

### Mediator assays

Histamine was measured by radioenzymatic assay and LTC_4_ by ELISA as described previously.^E1,E4^ The cytokines IL-5, IL-8, IL-13, and TNFα were measured by using a human T_H_1/T_H_2 multiplex plate on a Sector Imager 6000 (Meso Scale Discovery, Gaithersburg, Md).

### Allergen-induced bronchial smooth muscle contraction in isolated human bronchus

Lung tissue was obtained postmortem. Airways were dissected free of lung parenchyma and adjoining blood vessels. Secondary and tertiary bronchus, with cartilaginous walls and diameters of 3 to 10 mm, were cut spirally into strips approx 3 to 5 mm wide and then cut into pieces approximately 10 to 15 mm long. The strips were then passively sensitized overnight at room temperature (21°C) in atopic serum (20% v/v) in Krebs buffer. Before use, sensitized tissues were washed free of serum. Tissues were mounted under 1.5 g of resting tension in an immersion organ bath, maintained in oxygenated Krebs buffer solution at 37°C, and allowed to equilibrate for 30 to 45 minutes with 2 washes and retensioning if required.

Two preliminary “priming” contractions to 10 μM methacholine (Sigma, Poole, United Kingdom) were performed. The tissue was then incubated with Synta-66 (10 μM) or DMSO control (0.1% final concentration) for 1 hour. Grass allergen (Six grass mix, Alk-Abello, Hungerford, United Kingdom) was then added (0.1-30 U/mL final concentration), with contractions measured in milligrams tension. This was followed by the measurement of contraction to 10 μM methacholine. Data were expressed as % of the initial 10 μM methacholine contraction. Only limited quantities of viable human bronchus tissue were available, which allowed examination of the effects of only a single CRACM-channel blocker.
